# Validation of a Laboratory Method for Evaluating Dynamic Properties of Reconstructed Equine Racetrack Surfaces

**DOI:** 10.1371/journal.pone.0050534

**Published:** 2012-12-05

**Authors:** Jacob J. Setterbo, Anh Chau, Patricia B. Fyhrie, Mont Hubbard, Shrini K. Upadhyaya, Jennifer E. Symons, Susan M. Stover

**Affiliations:** 1 Biomedical Engineering Graduate Group, University of California Davis, Davis, California, United States of America; 2 J. D. Wheat Veterinary Orthopedic Research Laboratory, University of California Davis, Davis, California, United States of America; 3 Department of Mechanical and Aerospace Engineering, University of California Davis, Davis, California, United States of America; 4 Department of Biological and Agricultural Engineering, University of California Davis, Davis, California, United States of America; University of Liverpool, United Kingdom

## Abstract

**Background:**

Racetrack surface is a risk factor for racehorse injuries and fatalities. Current research indicates that race surface mechanical properties may be influenced by material composition, moisture content, temperature, and maintenance. Race surface mechanical testing in a controlled laboratory setting would allow for objective evaluation of dynamic properties of surface and factors that affect surface behavior.

**Objective:**

To develop a method for reconstruction of race surfaces in the laboratory and validate the method by comparison with racetrack measurements of dynamic surface properties.

**Methods:**

Track-testing device (TTD) impact tests were conducted to simulate equine hoof impact on dirt and synthetic race surfaces; tests were performed both *in situ* (racetrack) and using laboratory reconstructions of harvested surface materials. Clegg Hammer *in situ* measurements were used to guide surface reconstruction in the laboratory. Dynamic surface properties were compared between *in situ* and laboratory settings. Relationships between racetrack TTD and Clegg Hammer measurements were analyzed using stepwise multiple linear regression.

**Results:**

Most dynamic surface property setting differences (racetrack-laboratory) were small relative to surface material type differences (dirt-synthetic). Clegg Hammer measurements were more strongly correlated with TTD measurements on the synthetic surface than the dirt surface. On the dirt surface, Clegg Hammer decelerations were negatively correlated with TTD forces.

**Conclusions:**

Laboratory reconstruction of racetrack surfaces guided by Clegg Hammer measurements yielded TTD impact measurements similar to *in situ* values. The negative correlation between TTD and Clegg Hammer measurements confirms the importance of instrument mass when drawing conclusions from testing results. Lighter impact devices may be less appropriate for assessing dynamic surface properties compared to testing equipment designed to simulate hoof impact (TTD).

**Potential Relevance:**

Dynamic impact properties of race surfaces can be evaluated in a laboratory setting, allowing for further study of factors affecting surface behavior under controlled conditions.

## Introduction

Racetrack surface (layered material structure) is considered a risk factor for racehorse injuries and fatalities, but epidemiologic evidence, likely confounded by simultaneous risk factors, is contradictory providing no clear direction for desirable racetrack surface composition [1]. Since 2006 several California racetracks replaced their dirt surface with a synthetic surface in an attempt to reduce racehorse attrition. Despite data that supports a reduction in California fatality incidence with conversion from dirt to synthetic surfaces [1], the use of synthetic surfaces has been highly contested within the racehorse community. Management of synthetic surfaces has been challenging, leading to a debate of the costs and benefits of synthetic surfaces. Standardized, objective evaluations of equine racetrack surfaces and relevant confounding factors are needed to resolve this debate.

Initially, research examining race surfaces used subject-based (racehorse) methods, including observational studies [2] and instrumented horse measurements [3]–[8]. In addition to expense, instrumented racehorse studies are subject to large inter-horse variability [4], [7]. Simulated hoof impact testing devices [9]–[11] were developed to provide standardized, objective race surface measurements. However, results collected from these devices unearthed a subset of environmental confounding factors, such as moisture content [3], temperature [12], and maintenance [11]. Race surface measurements in a controlled, laboratory setting may elucidate the relevance and influence of such factors.

Equine race surface dynamic properties have been evaluated in controlled laboratory settings [9], [13]. One study determined boundary conditions for surface reconstruction in the laboratory [9]. However, none of these laboratory methods were validated to reproduce measurements obtained in the field. Validation of laboratory methods with respect to reproducibility of *in situ* (racetrack) measurements is needed to establish the value of laboratory measurements. Validation would allow for future studies of surface behavior and associated environmental factors in a controlled, laboratory setting.

The objectives of this study were to develop a method for laboratory reconstruction of equine race surfaces and to validate this method by comparison with *in situ* surface dynamic properties.

## Methods

### Study Design

A track-testing device (TTD) [9] that simulates hoof impact was used to measure dynamic properties of dirt and synthetic race surfaces *in situ* (2 racetracks, 4 days per surface) and after reconstruction of harvested surface materials in a laboratory box (3 experiments per surface). Clegg Hammer deceleration measurements at the racetracks guided laboratory surface reconstruction. Surface horizontal properties were assessed using a shear vane tester. Laboratory and racetrack measurements were compared, as well as, the relationships between Clegg Hammer and TTD measurements.

### Testing Equipment

#### TTD

**Figure 1:**
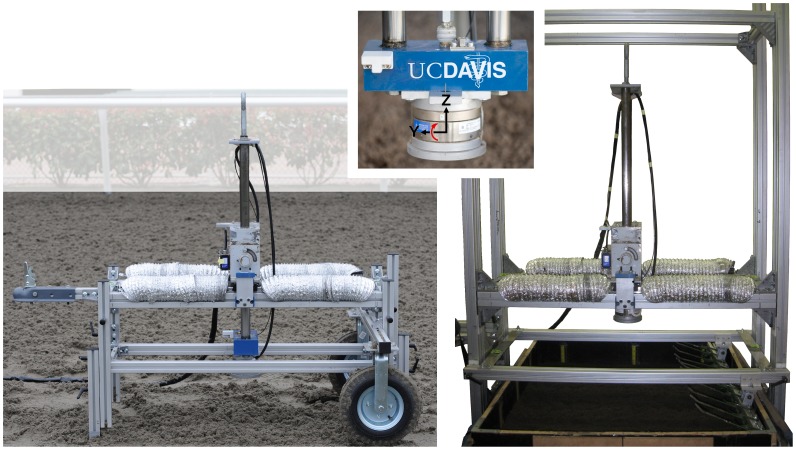
TTD shown on portable frame (left) and laboratory frame (right). The portable frame was leveled and fixed to the ground using 3 adjustable legs. The Z-axis (center) is parallel to the linear shafts (positive up), the X-axis is forward-backward (positive into page), and the Y-axis is lateral (positive left).

The TTD [9], [10] was attached to a portable frame that was placed on top of the surface at the racetracks and to a fixed frame in the laboratory (Figure 1). The TTD dropped a 27.8 kg, 12.7 cm diameter mass from 20.3, 30.5, and 40.6 cm to simulate the effective mass, surface area, and impact velocity of the hoof during fast trot and slow gallop [9]. Forces and linear displacement were collected at 2 kHz using custom software (LabVIEW, National Instruments Corp., Austin, TX).

#### Clegg hammer

**Figure 2:**
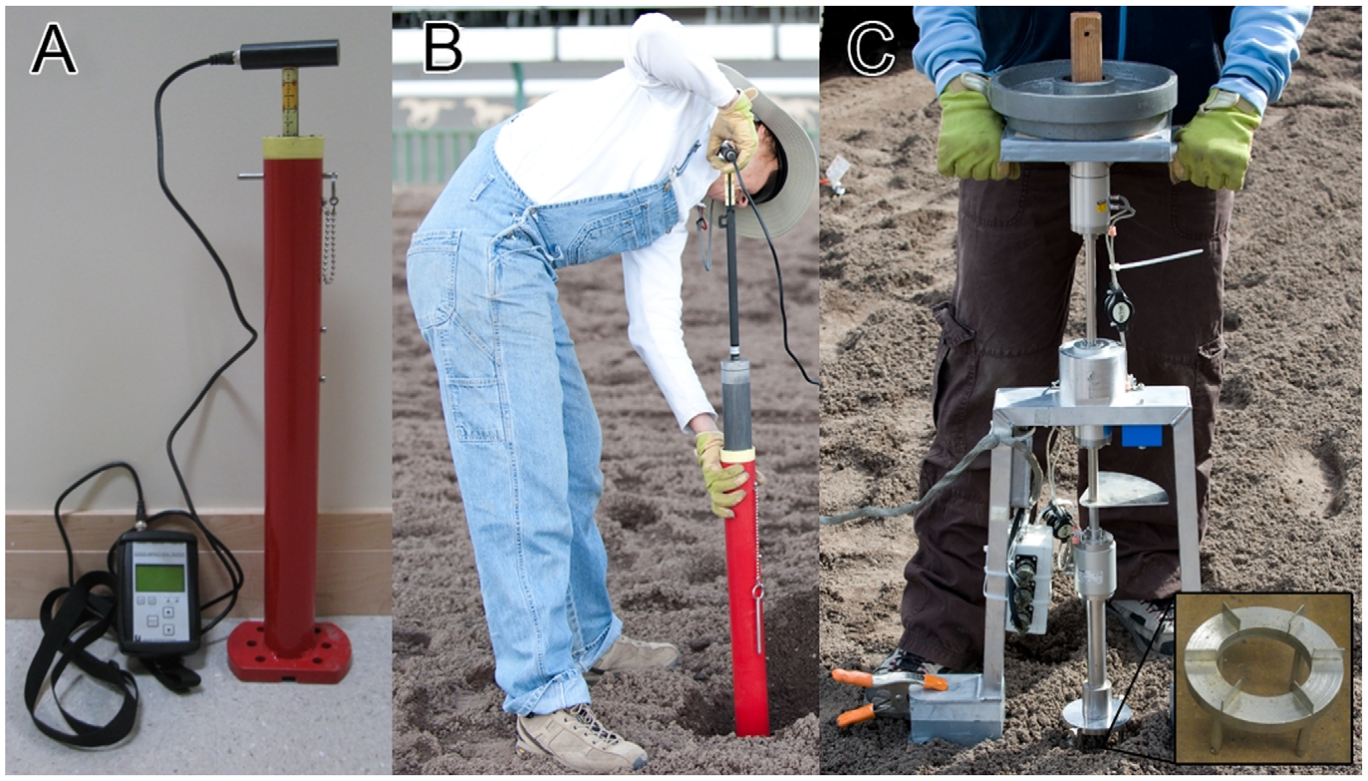
AB, Clegg Hammer. C, Shear vane device with weights applied. The shear Vane is rotated slowly until the sample fails to estimate the shear strength.

A 4.5 kg, 5 cm diameter cylindrical Clegg Hammer (Figure 2AB, Model 95050A, Lafayette Instrument Co., Lafayette, IN) was used to assess surface compaction. This device was portable, standardized [14], and previously implemented at racetracks. Maximum deceleration was measured in Clegg Impact Values (CIV, equivalent to 10 *g*). Residual deformation, the resting deformation relative to the pre-impact surface height (baseline, reference) was measured using a ruler (0.32 cm (0.125 in) resolution) attached to the Clegg Hammer handle.

#### Shear vane

A custom shear vane tester (Figure 2C, DIK-5502 SR-2 Type, Daiki Rika Kogyo Co., Konosu, Saitama, Japan) with normal force and torque load cells (MBA500 and QMA112, FUTEK, Irvine, CA) was used to assess surface horizontal properties. The shear interface was a stainless steel ring (6 cm inner diameter, 10 cm outer diameter) with 12 equally-spaced 2 cm wide by 1 cm high by 1 mm thick rectangular grousers. Weights were applied to adjust normal stress (10–40 kN/m^2^). Force and torque data were collected at 10 Hz. Force and torque data were converted to normal stress (σ) and shear stress (τ) by dividing by the surface area of soil affected by the shear interface.

### Racetracks and Race Surface Materials

California racetracks were tested in October and early November with surface preparations similar to those used for racehorse training. The dirt surface was watered and harrowed 8.3–8.9 cm deep, and the synthetic surface was harrowed 5 cm deep, but was not watered. The dirt racetrack had been recently converted to a “winter track,” with 2.3% organic matter and soil composition (UC Davis Analytical Laboratory, University of California, Davis, CA) of 83% sand, 10% silt, and 7% clay. The synthetic surface material was a proprietary wax-coated blend of sand (approximately 80% of composition), rubber, synthetic fibers and 15.7% organic matter. Surface material (∼380 L) was harvested in 4 layers by depth (2 inches each) at a single location from each racetrack for surface reconstruction in the laboratory.

### Racetrack Tests

TTD and Clegg Hammer tests were conducted at 3–5 locations/day for 4 days on each racetrack, starting on the homestretch (near the finish line), then 2–4 backstretch locations, and when possible, ending at a new location on the homestretch. At each location, 2 TTD impacts (initial, repeat) were performed at 6 spots spread over 7–10 m. The first 3 spots were vertical impacts with 20.3, 30.5, and 40.6 cm TTD drop distances, respectively; the next 3 spots were angled (linear shafts at 20° angle from vertical) impacts.

Clegg measurements (5 consecutive 45 cm drops) were taken at 4 depths (0, 5, 10, 15 cm) per location at individual spots within 1–3 m of a TTD spot.

Surface material temperature was measured with an infrared pen thermometer (Model 800100, Sper Scientific, Scottsdale, AZ) at each test location, at each depth (4 layers). Moisture content surface material samples (100–250 g) were obtained from the uppermost layer at all locations, and from depths 5, 10, and 15 cm below the uppermost layer from at least one location per day. Moisture content was determined using a modification of an ASTM standard [15]. The sample was dried to a constant mass in an oven at 65°C to prevent destroying synthetic surface material components [10]. Moisture content was calculated as mass lost during drying divided by mass of the dried sample.

Shear vane tests were performed at 1–2 locations/day. For each test, the shear vane was placed at a new harrowed location, a weight was applied, and the shear vane was rotated approximately 100° in 30–60 seconds. The 3 weights (2.3, 6.8, 11.3 kg) were each tested twice in random order at each location.

### Laboratory Surface Reconstruction

Race surfaces were reconstructed in the laboratory track-in-a-box, a 101.6 cm×101.6 cm (*width×length*) wood laboratory box with a base layer designed for a synthetic surface [9]. A boundary width of at least 90 cm and a surface depth of at least 20 cm were previously determined [9] to be large enough to avoid significant box boundary effects on impact measurements.

**Figure 3:**
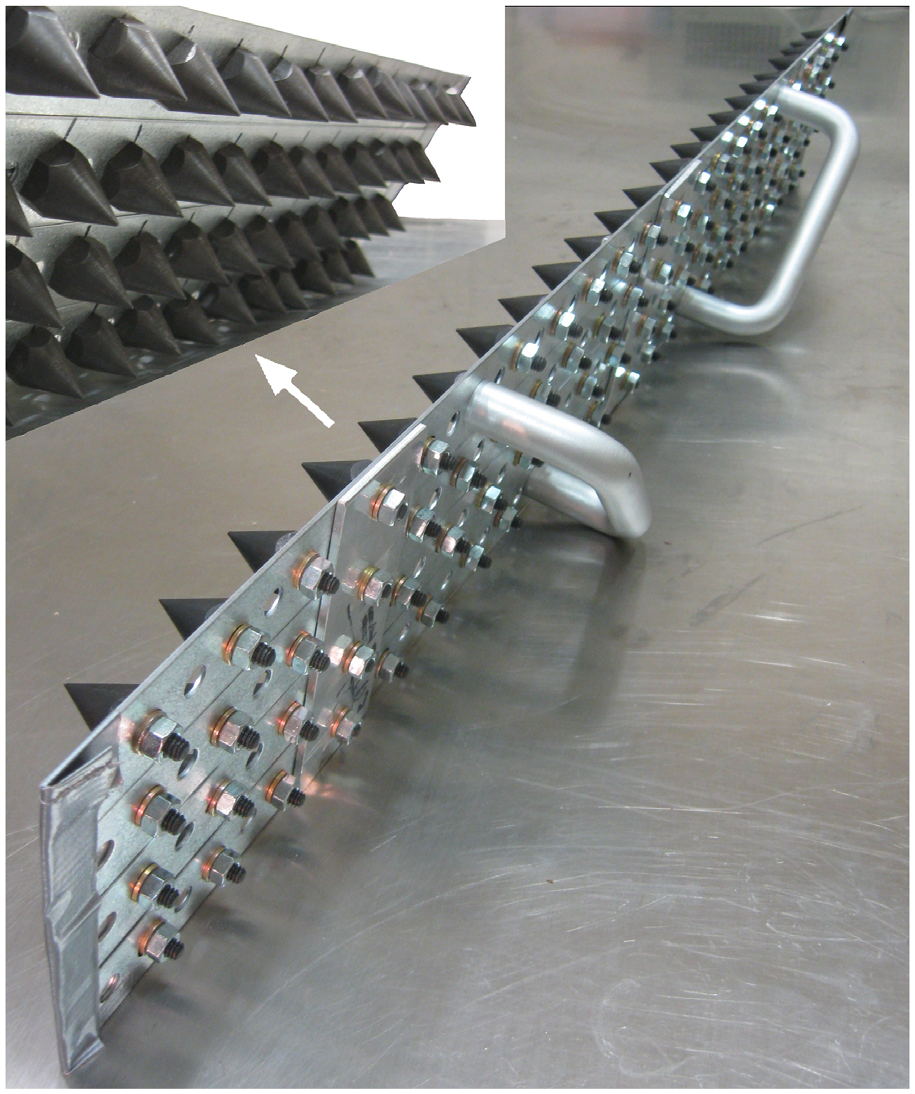
Custom compaction device. Conical spikes (92) were 1.9 cm wide, 2.5 cm high, and spaced 3.5 cm apart.

Three experiments were performed for both the dirt and synthetic surfaces. For each experiment, race surface material was reconstructed in layers on top of the plastic-covered asphalt of the laboratory box base. Layers were reconstructed using surface material from corresponding layers from the racetracks. For each layer in the laboratory box, a 2.5–5 cm depth of loose surface material was added, leveled, and compacted. Surface material was compacted using a combination of: a vibrating plate compactor (HULK ELECTRO, Evolution Power Tools, Davenport, IA), a manual impact-driven custom compaction device (Figure 3), a 95 cm long metal wedge, and a 2×5×95 cm wood board. Compaction was achieved by applying indentations using pressure and impacts with the board, metal wedge, and custom compaction device. Then the vibrating plate was moved back and forth across the surface several times with care not to excavate surface material. The procedure was repeated while reversing direction of plate movement to ensure compaction consistency. The reconstruction method was developed after evaluating various compaction methods during preliminary testing.

Compaction of each surface material layer was repeated until multiple Clegg measurements at 2–4 box locations were consistent and at least one 2^nd^ drop impact deceleration (CIV2) was within 10–20% of the racetrack average for each layer. CIV2 was chosen as the target value because the 1^st^ drop (CIV1) was often a seating drop, and CIV1 was often immeasurable at the racetracks (4.5 kg Clegg Hammer cannot read CIV<2.6). Racetrack data regression analyses showed upper layers were more correlated to TTD values, compared to lower layers (Table 1). Therefore, the lowest depth (15 cm) was often constructed harder in the laboratory than the racetracks, so that upper layers could achieve the correct hardness.

**Table 1:** Stepwise multiple linear regression results that correlated initial impact (40.6 cm vertical drop distance) TTD dynamic surface properties and Clegg Hammer measurements (CIV = impact deceleration, DEF = residual deformation) at the racetracks.

		Z-force									
		Maximum (N)		Average load rate (N/s)		Average stiffness (N/m)		Max load rate (N/s)		Max stiffness (N/m)	
Depth (cm)	Candidate Variable	Dirt	Synthetic	Dirt	Synthetic	Dirt	Synthetic	Dirt	Synthetic	Dirt	Synthetic
	**Intercept**	12,618	*1,325*	664,485	−708,194	274,848	−265,972	2,516,794	−1,552,357	683,867	−866,498
**0**	**CIV1 (CIV)**	NA	NA	NA	NA	NA	NA	NA	NA	NA	NA
	**CIV2 (CIV)**	ns	ns	ns	52,216	ns	21,642	ns	ns	ns	ns
	**CIV3 (CIV)**	ns	ns	ns	ns	ns	ns	ns	ns	ns	ns
	**DEF1 (cm)**	ns	ns	ns	ns	ns	ns	ns	ns	ns	ns
	**DEF3-DEF1 (cm)**	ns	**3,709**	ns	**420,406**	ns	**167,679**	ns	1,220,157	ns	504,390
**5**	**CIV1 (CIV)**	−**547**	NA	−**41,427**	NA	−**17,337**	NA	−**175,303**	NA	ns	NA
	**CIV2 (CIV)**	ns	644	ns	80,240	ns	30,694	ns	**315,349**	ns	ns
	**CIV3 (CIV)**	NA	ns	NA	ns	NA	ns	NA	ns	NA	**184,649**
	**DEF3-DEF1 (cm)**	ns	ns	ns	203,272	ns	72,281	ns	ns	ns	ns
**10**	**CIV1 (CIV)**	ns	NA	ns	NA	ns	NA	ns	NA	ns	NA
	**CIV2 (CIV)**	ns	ns	ns	ns	ns	ns	ns	ns	ns	ns
	**CIV3 (CIV)**	NA	ns	NA	ns	NA	ns	NA	ns	NA	ns
	**DEF3-DEF1 (cm)**	ns	ns	ns	ns	ns	ns	ns	ns	**631,615**	ns
**15**	**CIV1 (CIV)**	ns	ns	ns	ns	ns	ns	ns	ns	ns	ns
	**CIV2 (CIV)**	ns	ns	ns	ns	ns	ns	ns	ns	ns	ns
	**CIV3 (CIV)**	NA	NA	NA	NA	NA	NA	NA	NA	NA	NA
	**DEF3-DEF1 (cm)**	ns	ns	ns	ns	ns	ns	ns	ns	ns	ns
	**Adjusted R^2^**	0.23	0.67	0.34	0.88	0.39	0.84	0.27	0.73	0.28	0.53

Each column includes the stepwise regression coefficient for candidate variables (in rows), and adjusted R^2^ value for each model. **Bolded** terms had the strongest correlation for that model. For each depth of surface, the first 2 drops without missing CIV values were included as potential candidate variables. NA = not applicable or not tested due to missing values. ns = not significant (P>0.05). *Intercept* for synthetic vertical force maximum was not significant (P = 0.29).

Before adding a new surface layer, the compacted surface layer was lightly scraped or indented to help the layers bond. Instantaneous moisture content measurements were not available, so water was added periodically to account for drying and maintain consistent moisture content or to aid in compaction of lower layers.

**Figure 4:**
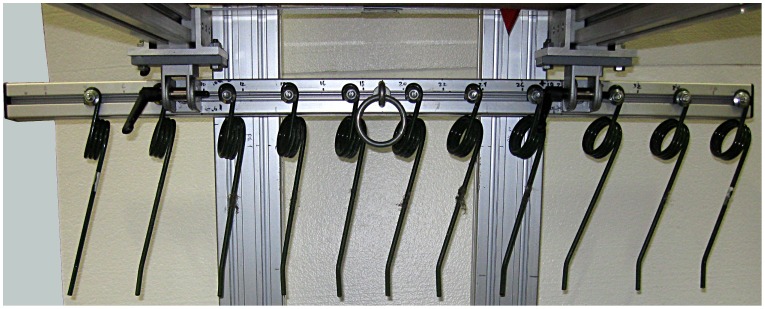
Harrow system.

Once the harder, lower layers were constructed to the desired height (14 cm for dirt, 15.2 cm for synthetic), the softer cushion layer was added to bring the surface height to 20.3 cm. Harrow methods were derived from knowledge of racetrack methods and equipment, and from preliminary testing results. Surfaces were harrowed by spring tines (Figure 4) with depths of 7.5 cm and 5 cm for the dirt and synthetic surfaces, respectively. The surface was leveled, and the top 1–2.5 cm were manually harrowed with a single tine to match the cushion softness of the racetracks. The dirt surface was harrowed in 5–10 cm spaced lines with a circular motion between the lines. The synthetic surface was harrowed similarly, except it was preceded by 2 cm wide linear depressions, spaced 5–8 cm apart, perpendicular to the harrow direction. After adding the cushion layer, several 40.6 cm vertical TTD impacts alternated with harrowing were performed to ensure that surface properties had stabilized and the harrow method used was satisfactory.

### Laboratory Tests

The laboratory portion of the study was designed as a split-plot ANOVA, where alpha = 0.05, SD of 0.05, and detectable contrast of 0.1 (twice as much as the SD). A t-test was used to compare laboratory and track differences. For each of the 3 experiments (reconstructions) for the dirt and synthetic surfaces, 2 sets of 6 TTD impacts (2 impact angles, each from 3 drop distances) were performed in the same randomized order as performed at each racetrack. The upper portion of the surface was harrowed after every impact. A single 40.6 cm vertical impact was performed between sets to check the stability of the values.

Shear vane tests were performed during the third experiment for each surface. For each test, the shear vane was placed on a harrowed location in the laboratory box and rotated approximately 110° in 90–150 seconds. The 3 weights (2.3, 6.8, 11.3 kg) were each tested 4 times in random order for each surface reconstruction.

Moisture content was measured for surface material samples from each layer (0, 5, 10, and 15 cm) at the end of each experiment. For the dirt surface, moisture content was measured at 0 and 5 cm below the top of the surface at the beginning and middle of the experiments to verify that moisture content was maintained as moisture was added periodically to replace moisture loss to the environment.

### Data Reduction

#### TTD data

Maxima, minima, and other attributes of force, moment, and displacement were extracted from data using custom software (MATLAB, The MathWorks Inc., Natick, MA). Zero force was established as average force over 0.1 s during free fall. A threshold vertical force of 40 N was used to determine times of impact initiation, termination, and duration. Impact was defined as the period of time when vertical force exceeded 40 N. Extracted data include impulse (integral of force with respect to time during impact), time to maximum force (time from impact initiation to peak force), average load rate (maximum force divided by time to maximum force), average rebound rate (maximum force divided by the time from peak force to impact termination), average stiffness (maximum force divided by maximum deformation), maximum load rate (maximum slope of force versus time, slope calculated from least squares fit of 3 points, or 0.0015 s), maximum rebound rate (minimum slope of force versus time) and maximum stiffness (maximum slope of force versus displacement).

Zero deformation was established as the position at impact initiation [9]. Elastic deformation (the distance the surface rebounds upward) was calculated by subtracting deformation at impact termination from maximum deformation. Similar calculations were used to determine plastic deformation (maximum deformation minus elastic deformation) and residual deformation (final motionless deformation).

Velocity was calculated from the time derivative of a quadratic fit of displacement versus time during free fall, and after impact. Impact and rebound velocities were the velocities at impact initiation and termination, respectively.

Total mechanical energy at impact initiation and termination were calculated as the sum of kinetic (*mv* ^2^/2) and potential energy (*mgh*) at respective time points. The potential energy datum was defined as the height at maximum deformation. Energy returned to the TTD (%) was defined as energy at impact termination divided by energy at impact initiation.

#### Shear vane data

Force and torque data were converted to normal stress (σ) and shear stress (τ) by dividing by the surface area that was compressed and sheared, respectively, by the shear interface. Values of maximum shear stress (τ_max_) and corresponding normal stress were fitted linearly to solve for cohesion (c) and angle of internal friction (φ) in the Mohr-Coulomb equation (τ_max_ = c+σ tan φ) [16].

### Racetrack TTD and Clegg Hammer Data Relationships

Relationships between racetrack TTD dynamic surface properties and racetrack Clegg Hammer measurements for each surface at respective racetracks were examined using stepwise multiple linear regression. Regression data were produced from each location that TTD measurements, and Clegg Hammer measurements at all 4 depths, were available. TTD initial impact results from the 40.6 cm height vertical impact were correlated with Clegg Hammer measurements from drops 1–3 at each depth to focus on the original harrowed surface condition. TTD dependent variables that incorporate main force, rate, and distance measurements (Z-force maximum, average load rate, average stiffness, maximum load rate, and maximum stiffness) were used in the regression analysis. The Clegg Hammer cannot record impact values less than 2.6. Because of this, values were not recorded for some tests. For each surface, the first 2 Clegg Hammer impact decelerations without missing values were used in the analysis (Table 1). Because both surfaces were more uneven at the racetracks than in the laboratory, the difference in deformation between the 3^rd^ and 1^st^ drops was used in the analysis to minimize the effect of surface unevenness on the relationships.

The stepwise procedure was first used to select possible Clegg Hammer predictor variables (P<0.15 to enter, P>0.15 to remove) related to individual TTD variables (Table 1). Subsequently, any predictor variables that were not statistically significant (P≥0.05) were removed. The analysis was repeated until only statistically significant predictor variables and the intercept remained in the model. All final models were statistically significant (P<0.05).

### Laboratory-Racetrack Comparisons

Laboratory and racetrack TTD measurements were compared using a one-sample t-test on the differences between the laboratory value and racetrack average for each surface [10] (SAS, version 9.2, SAS Institute Inc., Cary, NC). For every laboratory measurement (N = 36 total impacts per surface), the setting difference (laboratory-racetrack, [ΔL,T]) was calculated by subtracting the racetrack initial impact average (average of 54 impacts from 3 days and 9 locations per surface [10]) from the laboratory measurement. The setting difference was also analyzed using a repeated measures mixed model ANOVA (SAS) to assess if the effects of surface type, impact velocity, impact angle, set (1^st^, 2^nd^), and all interactions significantly changed the magnitude of the setting difference. Finally, laboratory measurements were analyzed independently from *in situ* measurements using repeated measures mixed model ANOVA to assess the effect of surface, impact velocity, impact angle, set, and all interactions on TTD dynamic surface properties. Tests of the assumptions inherent in the statistical analyses were performed and met in the current study. For example, assessment of normality of the residuals from ANOVAs were examined. Those variables not exhibiting normality by the Shapiro-Wilk test were further assessed by histogram plots, which were determined to be normal.

## Results

Following layered hardness surface material layer reconstruction, laboratory dynamic properties closely approximated *in situ* (racetrack) measurements.

### Racetrack Regression Analysis

**Figure 5:**
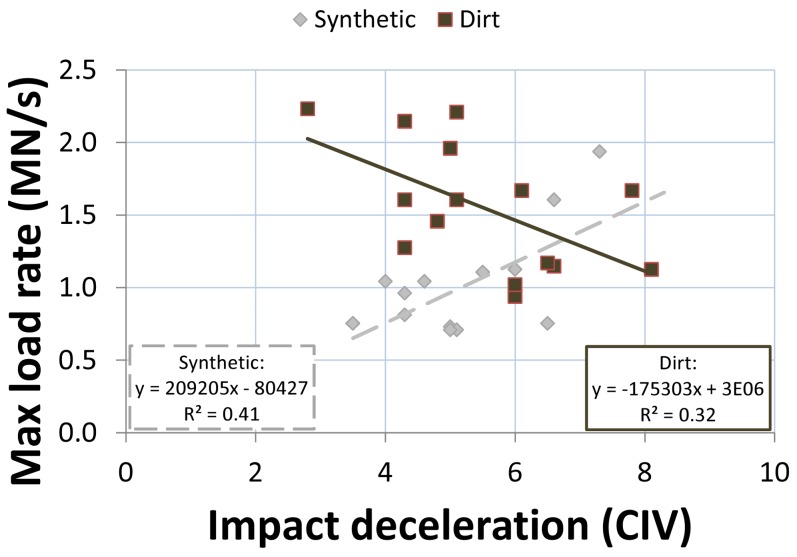
Example simple linear regression displaying TTD maximum load rate (MN/s, meganewton per second) versus Clegg Hammer impact deceleration (CIV, Clegg impact value) at 5 cm below the surface: CIV1 is plotted for the dirt surface and CIV2 for the synthetic surface.

Clegg Hammer-TTD correlations were stronger for the synthetic racetrack surface than for the dirt racetrack surface (Table 1). Clegg Hammer measurements accounted for 53–88% of the variability in TTD variables for the synthetic racetrack surface, but only 23–39% for the dirt racetrack surface. Clegg impact decelerations were positively correlated with higher forces and load rates for the synthetic racetrack surface, but negatively correlated for the dirt racetrack surface (Figure 5). For both racetrack surfaces, greater deformations at 5 or 10 cm depths were correlated with harder TTD dynamic racetrack surface characteristics. Shallower Clegg Hammer measurements (0, 5 cm) were more closely correlated with TTD values than deeper measurements (10, 15 cm).

### Laboratory-Racetrack Analysis

#### Moisture content

Laboratory dirt surface material moisture content measurements were slightly less (≤0.5%) than those at the racetrack except for the uppermost layer (0 cm), where the laboratory moisture content was 1.9% greater than the racetrack moisture content (Table 2). Laboratory synthetic surface material moisture content measurements were nearly identical (0–0.2% difference) to those from the racetrack at upper layers (0, 5 cm depths), but up to 5.5% greater than racetrack values at lower layers (10, 15 cm depths, Table 2).

**Table 2:** Racetrack and laboratory surface material moisture contents (%, percent) and temperatures (°, degree) [mean (standard deviation)] at 4 depths (cm, centimeter) measured from the top of the surface material.

	Moisture Content (%)				Temperature (°C)	
	Dirt		Synthetic		Dirt	Synthetic
Depth (cm)	Racetrack	Laboratory	Racetrack	Laboratory	Racetrack	Racetrack
**0**	8.8 (3.1)	10.7 (0.2)	0.2 (0.1)	0.2 (0.03)	20.9 (11.0)	27.6 (8.8)
**5**	10.7 (1.4)	10.4 (0.1)	0.3 (0.3)	0.5 (0.1)	15.7 (4.2)	24.1 (5.1)
**10**	11.0 (0.3)	10.7 (0.5)	0.2 (0.1)	5.7 (1.8)	15.5 (3.3)	21.7 (3.5)
**15**	10.8 (0.5)	10.3 (0.7)	6.0 (4.7)	9.4 (1.3)	15.1 (2.5)	19.8 (1.8)

Laboratory surface temperature was approximately 21.5°C.

#### Temperature

Laboratory ambient and surface material temperatures were approximately 21.5°C. Ambient temperatures were 13.3±4.0°C at the dirt racetrack and 18.1±6.1°C at the synthetic racetrack. Racetrack surface material temperatures were warmer than the average ambient temperature, with upper layers warmer than lower layers (Table 2).

#### Clegg hammer

Average laboratory 2^nd^ drop impact decelerations (CIV2) were 91–120% of racetrack averages (Table 3). Laboratory 2^nd^ drop residual deformations were 38–91% of racetrack averages. Laboratory differences in residual deformation between the 3^rd^ and 1^st^ drops (Table 3, Drop (3–1)) were 83–200% of racetrack averages. At deeper depths, the racetrack averages showed that after 3 drops decelerations and deformations do not change greatly, whereas upper depths continued to get harder between drops (Table 3).

**Table 3:** Dirt and synthetic Clegg Hammer impact decelerations (CIV, Clegg impact value) and residual deformations (cm, centimeter) (mean (STD)) at 4 depths (cm) measured from the top of the surface material *in situ* (racetrack) and in the laboratory.

		Impact deceleration (CIV)				Residual deformation (cm)				
		Dirt		Synthetic		Dirt		Synthetic		
Depth (cm)	Drop	Racetrack	Laboratory	Racetrack	Laboratory	Racetrack	Laboratory	Racetrack	Laboratory	
**0**	**1**	3.1 (0.4)	3.6 (NA)	3.1 (0.5)	2.5 (NA)	4.1 (0.3)	3.5 (0.3)	2.5 (0.6)	2.2 (0.5)	
	**2**	6.4 (1.4)	7.0 (0.5)	4.3 (0.7)	4.3 (0.3)	4.3 (0.3)	3.9 (0.4)	2.9 (0.7)	2.5 (0.5)	
	**3**	8.2 (2.5)	8.8 (0.7)	5.1 (0.7)	5.0 (0.6)	4.5 (0.3)	4.3 (0.2)	3.3 (0.7)	3.0 (0.4)	
	**4**	10.9 (1.9)	NA	5.7 (0.6)	NA	4.7 (0.2)	NA	3.5 (0.7)	NA	
	**5**	11.8 (1.9)	NA	5.9 (0.7)	NA	4.8 (0.3)	NA	3.8 (0.8)	NA	
	**(3–1)**	NA	NA	NA	NA	0.4 (0.2)	0.8 (0.2)	0.8 (0.2)	0.7 (0.2)	
**5**	**1**	5.1 (1.4)	7.8 (1.3)	3.8 (0.8)	4.4 (0.6)	1.9 (0.5)	0.6 (0.0)	1.3 (0.3)	0.6 (0.3)	
	**2**	9.2 (1.2)	10.5 (0.9)	5.2 (1.0)	5.5 (0.3)	2.3 (0.5)	1.0 (0.0)	1.7 (0.3)	1.0 (0.3)	
	**3**	11.5 (1.2)	11.2 (0.8)	5.8 (0.9)	6.0 (0.6)	2.5 (0.4)	1.2 (0.2)	2.0 (0.4)	1.3 (0.3)	
	**4**	12.3 (1.3)	NA	6.2 (0.9)	NA	2.7 (0.5)	NA	2.3 (0.4)	NA	
	**5**	13.1 (1.6)	NA	6.5 (0.9)	NA	2.9 (0.5)	NA	2.5 (0.4)	NA	
	**(3–1)**	NA	NA	NA	NA	0.6 (0.1)	0.5 (0.2)	0.6 (0.3)	0.6 (0.0)	
**10**	**1**	13.7 (1.3)	12.7 (1.4)	5.5 (0.9)	4.4 (0.3)	0.7 (0.1)	0.3 (0.0)	0.8 (0.2)	0.6 (0.0)	
	**2**	16.1 (1.4)	14.7 (0.8)	6.7 (0.7)	6.2 (0.3)	0.8 (0.2)	0.4 (0.2)	1.1 (0.2)	1.0 (0.0)	
	**3**	16.3 (1.5)	15.0 (0.3)	7.2 (1.0)	6.9 (0.4)	1.0 (0.2)	0.8 (0.4)	1.2 (0.3)	1.3 (0.0)	
	**4**	16.6 (1.3)	NA	7.7 (0.6)	NA	1.1 (0.2)	NA	1.5 (0.3)	NA	
	**5**	16.6 (1.2)	NA	7.8 (0.8)	NA	1.3 (0.3)	NA	1.7 (0.4)	NA	
	**(3–1)**	NA	NA	NA	NA	0.3 (0.2)	0.5 (0.4)	0.4 (0.2)	0.6 (0.0)	
**15**	**1**	14.9 (1.9)	15.8 (0.7)	7.1 (1.6)	7.8 (0.3)	0.7 (0.3)	0.3 (0.0)	1.1 (0.5)	0.3 (0.0)	
	**2**	17.9 (1.9)	18.9 (1.0)	10.0 (1.2)	12.0 (1.9)	0.8 (0.3)	0.3 (0.0)	1.2 (0.5)	0.6 (0.0)	
	**3**	18.3 (2.0)	20.3 (0.1)	10.5 (1.3)	13.6 (1.3)	0.8 (0.3)	0.6 (0.0)	1.4 (0.5)	0.8 (0.2)	
	**4**	18.1 (1.5)	NA	10.9 (1.5)	NA	1.0 (0.3)	NA	1.5 (0.5)	NA	
	**5**	18.3 (1.2)	NA	11.4 (2.1)	NA	1.1 (0.3)	NA	1.6 (0.5)	NA	
	**(3–1)**	NA	NA	NA	NA	0.2 (0.2)	0.3 (0.0)	0.3 (0.2)	0.3 (0.3)	

Laboratory values correspond to the set of Clegg Hammer measurements with the hardest CIV2 value. (3–1) = difference in residual deformation between drops 3 and 1. NA = Not applicable or not tested.

#### TTD

Impact velocities (mean±SD) for the 3 drop distances (20.3, 30.5, and 40.6 cm) were 1.85±0.05, 2.25±0.04, and 2.58±0.06 m/s at the racetracks, and 1.82±0.05, 2.22±0.05 and 2.53±0.05 m/s in the laboratory. On average, laboratory impact velocity was 0.03 m/s (1.3%) less and significantly different than racetrack impact velocity (P<0.001). Angle impact velocity was always less than the corresponding vertical impact velocity, 2% less at the racetracks and 4% less in the laboratory.

**Figure 6:**
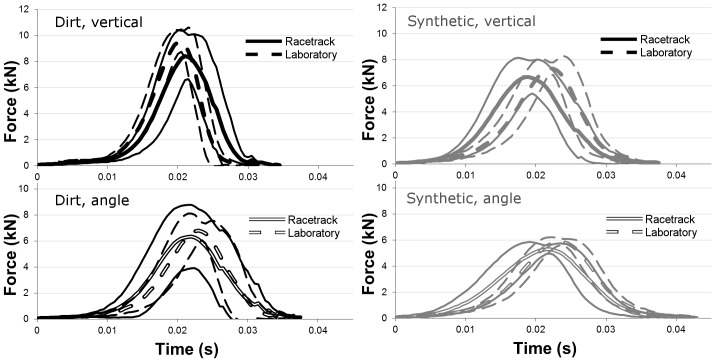
Mean force (kN, kilonewton) versus time (s, second) traces for all 40.6 cm vertical (top) and angled (bottom) TTD impact data for racetrack measurements (solid lines: thick, mean; thin, ± standard deviation) and laboratory measurements (dashed lines: thick, mean; thin, ± standard deviation).

Most TTD setting differences (ΔL,T) were 10% or less of the respective racetrack averages, and the differences were less than 6% for key values like Z-force maximum, average load rate, and average stiffness (Table 4). When compared to the respective *in-situ* surface type differences (dirt-synthetic) (ΔSurf_T_), the setting differences were less than 14% for these same key values (Table 4). The laboratory dirt surface reconstruction replicated the racetrack slightly better than the laboratory synthetic surface reconstruction (Figure 6). The dirt setting difference was significantly different than zero for 8 of the 22 dynamic surface properties, while the synthetic setting difference was significantly different for 16 parameters (Table 4). The synthetic setting difference was more than 10% of the racetrack average for more dynamic surface properties than for the dirt surface, including Z-force maximum stiffness, elastic deformation, and total mechanical energy returned to the TTD. The racetrack surface type difference (ΔSurf_T_) was very small for time-related and energy-related variables (e.g., impact duration, time to maximums, and energies). Consequently, the setting difference was a larger proportion of the racetrack surface type difference for these variables.

**Table 4:** TTD dynamic surface properties of initial vertical impact in the laboratory and *in situ* (racetrack).

				Laboratory vs. Racetrack							
	Racetrack			Dirt				Synthetic			
Variable	Mean_D_	Mean_S_	ΔSurf_T_	ΔL,T	Pvalue	ΔL,T/Mean_D_	ΔL,T/|ΔSurf_T_|	ΔL,T	P value	ΔL,T/Mean_S_	ΔL,T/|ΔSurf_T_|
Z-force											
maximum (kN)	7.29	5.33	**1.96**	−0.091	*0.321*	−**1%**	−**5%**	0.262	*<0.001*	**5%**	**13%**
impact duration (ms)	33.3	39.5	−**6.2**	0.34	*0.448*	**1%**	**5%**	1.79	*0.002*	**5%**	**29%**
time to maximum (ms)	23.8	22.8	**1.0**	−0.41	*0.241*	−**2%**	−**41%**	1.69	*<0.001*	**7%**	**169%**
impulse (N-s)	68	72	−**4.0**	−0.45	*0.022*	−**1%**	−**11%**	0.28	*0.089*	**0%**	**7%**
Z-force load rate											
average load rate (MN/s)	0.32	0.24	**0.08**	0.001	*0.887*	**0%**	**1%**	−0.008	*0.200*	−**3%**	−**10%**
max load rate (MN/s)	1.06	0.6	**0.46**	−0.021	*0.428*	−**2%**	−**5%**	0.077	*<0.001*	**13%**	**17%**
average rebound rate (MN/s)	0.81	0.34	**0.47**	−0.065	*0.002*	−**8%**	−**14%**	0.017	*0.107*	**5%**	**4%**
max rebound rate (MN/s)	2.34	0.99	**1.35**	−0.084	*0.146*	−**4%**	−**6%**	0.064	*0.015*	**6%**	**5%**
Z-force stiffness											
average stiffness (MN/m)	0.15	0.12	**0.03**	0.0027	*0.452*	**2%**	**9%**	−0.0025	*0.394*	−**2%**	−**8%**
max stiffness (MN/m)	0.73	0.39	**0.34**	0.019	*0.441*	**3%**	**6%**	0.089	*<0.001*	**23%**	**26%**
Deformation											
plastic (cm)	4.7	4.22	**0.48**	−0.155	*0.027*	−**3%**	−**32%**	0.246	*0.002*	**6%**	**51%**
elastic (cm)	0.15	0.37	−**0.22**	0.009	*0.026*	**6%**	**4%**	0.040	*<0.001*	**11%**	**18%**
maximum (cm)	4.85	4.58	**0.27**	−0.147	*0.036*	−**3%**	−**54%**	0.286	*<0.001*	**6%**	**106%**
time to maximum (ms)	26.8	27.2	−**0.4**	−0.22	*0.569*	−**1%**	−**55%**	1.53	*0.002*	**6%**	**383%**
residual (cm)	4.68	4.04	**0.64**	−0.143	*0.040*	−**3%**	−**22%**	0.209	*0.007*	**5%**	**33%**
Total mechanical energy											
at impact initiation (J)	82.4	82.5	−**0.1**	−2.28	*<0.001*	−**3%**	−**2280%**	−1.70	*<0.001*	−**2%**	−**1700%**
at impact termination (J)	1.5	2.7	−**1.2**	−0.05	*0.298*	−**3%**	−**4%**	0.36	*<0.001*	**13%**	**30%**
returned to TTD (%)	1.8	3.2	−**1.4**	0.02	*0.719*	**1%**	**1%**	0.49	*<0.001*	**15%**	**35%**
X-force											
minimum (kN)	0.34	0.25	**0.09**	0.004	*0.572*	**1%**	**4%**	0.037	*0.003*	**15%**	**41%**
time to minimum (ms)	21	16.1	**4.9**	−3.27	*0.017*	−**16%**	−**67%**	2.07	*0.173*	**13%**	**42%**
Y-moment											
minimum (N-m)	95.9	69.5	**26.4**	−4.06	*0.070*	−**4%**	−**15%**	10.13	*0.002*	**15%**	**38%**
time to minimum (ms)	19.4	17.5	**1.9**	0.96	*0.380*	**5%**	**51%**	2.58	*0.066*	**15%**	**136%**

Racetrack averages (average over all other factors) of the dirt (Mean_D_) and synthetic (Mean_S_) surfaces, and their difference (ΔSurf_T_ = Mean_D_−Mean_S_), were used to assess the magnitude of ΔL,T (average of laboratory value minus racetrack average for each property). If P<0.05 then ΔL,T is significantly different than zero. |ΔSurf_T_| = absolute value of the difference between dirt and synthetic racetrack averages (ΔSurf_T_).

Setting differences (ΔL,T) were significantly affected, but to a relatively small degree, by surface type, impact velocity, and impact angle for 3, 10, and 19 parameters, respectively (22 dynamic surface properties total, Table 5). Surface effects were inconsistent on setting differences. Impacts at 1.82 m/s velocity were consistently less stiff and impacts at 2.22 m/s velocity were consistently harder in the laboratory than in situ. But, impacts at 2.53 m/s velocity were inconsistent. Vertical impacts were consistently harder and angled impacts were consistently less stiff in the laboratory. Set number did not significantly affect any results.

**Table 5:** Adjusted least squares means of the laboratory-racetrack difference in TTD dynamic surface properties for each main effect surface, impact velocity [m/s, meter per second], impact angle [°, degree]) averaged over all other factors, and reported as a percentage of the overall racetrack average for that variable.

	Surface			Impact velocity				Impact angle		
Variable	Dirt	Synthetic	P value	1.82 m/s	2.22 m/s	2.53 m/s	P value	0°-Vertical	20°-Angle	P value
Z-force										
maximum (kN)	−1%	4%	*0.120*	−1%	3%	2%	*0.101*	5%	−2%	*<0.001*
impact duration (ms)	1%	5%	*0.364*	5%	−1%	5%	*0.003*	0%	6%	*<0.001*
time to maximum (ms)	−2%	7%	*0.136*	5%	−1%	4%	*0.042*	0%	5%	*0.005*
impulse (N-s)	−1%	0%	*0.006*	0%	0%	0%	*0.558*	1%	−1%	*<0.001*
Z-force load rate										
average load rate (MN/s)	0%	−3%	*0.655*	−4%	4%	−4%	*0.058*	4%	−6%	*<0.001*
max load rate (MN/s)	−3%	9%	*0.106*	−2%	5%	7%	*0.158*	9%	−2%	*0.008*
average rebound rate (MN/s)	−11%	3%	*0.165*	−5%	1%	−8%	*0.063*	2%	−10%	*<0.001*
max rebound rate (MN/s)	−5%	4%	*0.199*	−3%	1%	0%	*0.525*	6%	−8%	*<0.001*
Z-force stiffness										
average stiffness (MN/m)	2%	−2%	*0.580*	−3%	5%	−2%	*0.012*	5%	−5%	*<0.001*
max stiffness (MN/m)	3%	16%	*0.111*	−1%	12%	18%	*<0.001*	21%	−2%	*<0.001*
Deformation										
plastic (cm)	−3%	6%	*0.126*	3%	−3%	3%	*0.012*	−2%	4%	*0.005*
elastic (cm)	3%	15%	*0.016*	6%	7%	15%	*0.011*	8%	11%	*0.361*
maximum (cm)	−3%	6%	*0.106*	3%	−2%	4%	*0.007*	−1%	4%	*0.003*
time to maximum (ms)	−1%	6%	*0.206*	5%	−1%	4%	*0.018*	−1%	6%	*<0.001*
residual (cm)	−3%	5%	*0.166*	2%	−3%	3%	*0.013*	−2%	4%	*0.003*
Total mechanical energy										
at impact initiation (J)	−3%	−2%	*0.307*	−2%	−3%	−3%	*<0.001*	−2%	−3%	*<0.001*
at impact termination (J)	−2%	17%	*0.093*	6%	8%	9%	*0.788*	12%	2%	*0.001*
returned to TTD (%)	1%	20%	*0.123*	11%	10%	9%	*0.862*	15%	6%	*0.004*
X-force										
minimum (kN)	1%	12%	*0.157*	4%	7%	9%	*0.570*	−3%	17%	*<0.001*
time to minimum (ms)	−18%	11%	*0.140*	−14%	2%	2%	*0.345*	−11%	5%	*0.116*
Y-moment										
minimum (N-m)	−5%	12%	*0.031*	−1%	8%	4%	*0.089*	−1%	8%	*0.004*
time to minimum (ms)	5%	14%	*0.645*	7%	9%	13%	*0.923*	9%	10%	*0.972*

For each main effect, a P<0.05 means the magnitude of the laboratory-racetrack difference changed significantly when the level of that main effect changed.

When analyzing laboratory results alone, there were significant differences between surfaces for 14 of 22 dynamic surface properties considered (Table 6). Synthetic surface Z-force maximum, load rates, and stiffnesses were 46–78% of dirt surface values. Synthetic surface elastic deformation and total mechanical energy returned to the TTD were 256% and 206%, respectively, of dirt surface values. Impact velocity was significant for 19 of 22 dynamic surface properties (higher impact velocities generally resulted in stiffer properties), and impact angle was significant for all dynamic surface properties (vertical impacts generally had stiffer properties than angle impacts). Set number did not significantly affect any results.

**Table 6:** Laboratory TTD dynamic surface properties [adjusted least squares means (standard error)] for each main effect (surface, impact velocity [m/s, meter per second], impact angle [°, degree]) averaged over all other factors.

	Surface		Impact velocity			Impact angle	
Variable	Dirt	Synthetic	1.82 m/s	2.22 m/s	2.53 m/s	0°-Vertical	20°-Angle
Z-force							
maximum (kN)	7.20 (0.13)^a^	5.59 (0.13)^b^	4.75 (0.11)^a^	6.52 (0.11)^b^	7.93 (0.11)^c^	7.24 (0.10)^a^	5.56 (0.10)^b^
impact duration (ms)	33.7 (1.0)^a^	41.3 (1.0)^b^	40.9 (0.8)^a^	36.2 (0.8)^b^	35.4 (0.8)^b^	34.5 (0.7)^a^	40.5 (0.7)^b^
time to maximum (ms)	23.4 (0.8)^a^	24.5 (0.8)^a^	26.6 (0.6)^a^	22.9 (0.6)^b^	22.3 (0.6)^b^	23.0 (0.6)^a^	24.9 (0.6)^b^
impulse (N-s)	67.6 (0.1)^a^	72.3 (0.1)^b^	59.5 (0.1)^a^	70.5 (0.1)^b^	79.9 (0.1)^c^	72.0 (0.1)^a^	67.8 (0.1)^b^
Z-force load rate							
average load rate (MN/s)	0.32 (0.01)^a^	0.24 (0.01)^b^	0.18 (0.01)^a^	0.29 (0.01)^b^	0.36 (0.01)^c^	0.33 (0.01)^a^	0.23 (0.01)^b^
max load rate (MN/s)	1.04 (0.03)^a^	0.68 (0.03)^b^	0.55 (0.03)^a^	0.87 (0.03)^b^	1.16 (0.03)^c^	1.02 (0.03)^a^	0.70 (0.03)^b^
average rebound rate (MN/s)	0.74 (0.03)^a^	0.35 (0.03)^b^	0.39 (0.03)^a^	0.57 (0.03)^b^	0.69 (0.03)^c^	0.70 (0.03)^a^	0.40 (0.03)^b^
max rebound rate (MN/s)	2.26 (0.07)^a^	1.05 (0.07)^b^	1.13 (0.06)^a^	1.68 (0.06)^b^	2.15 (0.06)^c^	2.19 (0.05)^a^	1.12 (0.05)^b^
Z-force stiffness							
average stiffness (MN/m)	0.15 (0.01)^a^	0.11 (0.01)^b^	0.11 (0.01)^a^	0.14 (0.01)^b^	0.15 (0.01)^c^	0.16 (0.01)^a^	0.11 (0.01)^b^
max stiffness (MN/m)	0.75 (0.02)^a^	0.48 (0.02)^b^	0.48 (0.02)^a^	0.63 (0.02)^b^	0.73 (0.02)^c^	0.76 (0.02)^a^	0.47 (0.02)^b^
Deformation							
plastic (cm)	4.54 (0.15)^a^	4.46 (0.15)^a^	4.19 (0.12)^a^	4.36 (0.12)^a^	4.96 (0.12)^b^	4.35 (0.11)^a^	4.66 (0.11)^b^
elastic (cm)	0.16 (0.01)^a^	0.41 (0.01)^b^	0.23 (0.01)^a^	0.29 (0.01)^b^	0.33 (0.01)^c^	0.27 (0.01)^a^	0.30 (0.01)^b^
maximum (cm)	4.70 (0.15)^a^	4.87 (0.15)^a^	4.42 (0.12)^a^	4.64 (0.12)^b^	5.29 (0.12)^c^	4.61 (0.11)^a^	4.96 (0.11)^b^
time to maximum (ms)	26.6 (0.8)^a^	28.7 (0.8)^a^	30.6 (0.7)^a^	26.4 (0.7)^b^	25.9 (0.7)^b^	25.9 (0.6)^a^	29.4 (0.6)^b^
residual (cm)	4.54 (0.15)^a^	4.25 (0.15)^a^	4.10 (0.12)^a^	4.25 (0.12)^a^	4.83 (0.12)^b^	4.21 (0.11)^a^	4.57 (0.11)^b^
Total mechanical energy							
at impact initiation (J)	80.2 (0.4)^a^	80.8 (0.4)^a^	57.5 (0.3)^a^	80.7 (0.3)^b^	103.2 (0.3)^c^	83.0 (0.3)^a^	77.9 (0.3)^b^
at impact termination (J)	1.4 (0.1)^a^	3.0 (0.1)^b^	1.6 (0.1)^a^	2.3 (0.1)^b^	2.9 (0.1)^c^	2.6 (0.1)^a^	1.9 (0.1)^b^
returned to TTD (%)	1.8 (0.2)^a^	3.7 (0.2)^b^	2.7 (0.1)^a^	2.8 (0.1)^a^	2.8 (0.1)^a^	3.1 (0.1)^a^	2.4 (0.1)^b^
X-force							
minimum (kN)	0.34 (0.01)^a^	0.28 (0.01)^b^	0.24 (0.01)^a^	0.32 (0.01)^b^	0.38 (0.01)^c^	0.04 (0.01)^a^	0.59 (0.01)^b^
time to minimum (ms)	17.8 (2.1)^a^	18.2 (2.1)^a^	17.1 (1.9)^a^	19.0 (1.9)^a^	17.8 (1.9)^a^	13.1 (1.7)^a^	22.9 (1.7)^b^
Y-moment							
minimum (N-m)	91.8 (3.1)^a^	79.6 (3.1)^b^	66.3 (2.8)^a^	90.1 (2.8)^b^	100.7 (2.8)^c^	6.7 (2.5)^a^	164.7 (2.5)^b^
time to minimum (ms)	20.3 (2.3)^a^	20.1 (2.3)^a^	20.8 (2.3)^a^	19.3 (2.3)^a^	20.5 (2.3)^a^	14.4 (2.0)^a^	26.0 (2.0)^b^

Dynamic surface properties within a variable row and within each main effect with different superscripts are significantly (P<0.05) different. Main effects were significant unless values are underlined.

#### Shear vane

**Figure 7:**
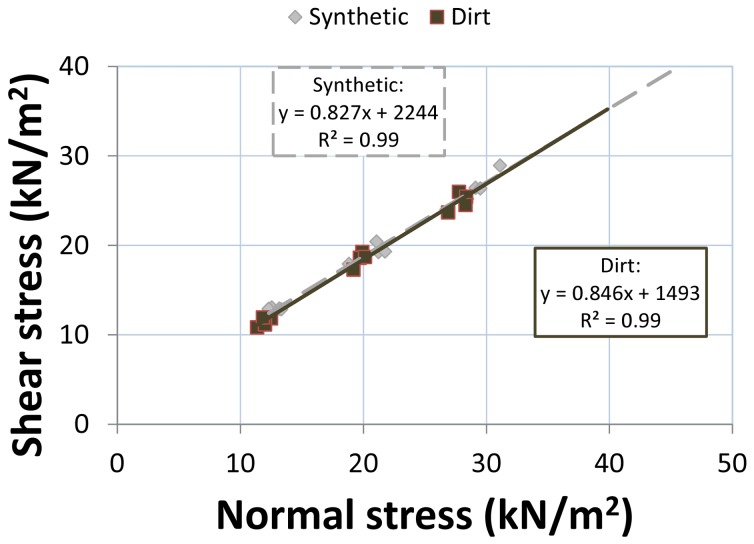
Maximum shear stress (kN/m^2^, kilonewton per square meter) versus corresponding normal stress for the dirt and synthetic surface at the laboratory. Results of linear fit were used to find cohesion and angle of internal friction.

Shear stresses ranged from 10–29 kN/m^2^ from 12 tests per surface at the laboratory (Figure 7), and from 9–37 kN/m^2^ from 36 tests per surface at the racetracks [10]. Laboratory shear vane results were more similar to racetrack results for the synthetic surface than for the dirt surface (Table 7). Assuming the Mohr-Coulomb failure criterion holds for a normal stress of 400 kN/m^2^ (5,000 N force applied over 0.0125 m^2^ area, approximating trotting racehorse normal stress), synthetic shear failure stress was 5% greater at the laboratory than at the racetrack, whereas dirt surface shear failure stress was 27% greater. At a normal stress of 400 kN/m^2^, the laboratory dirt surface shear failure stress was 2% greater than the laboratory synthetic surface value.

**Table 7:** Racetrack and laboratory cohesion (c, N/m^2^, newton per square meter), angle of internal friction (φ, °, degree), and R^2^ from the linear fit of shear vane data, and shear failure stress at a normal stress of 400 kN/m^2^ (τ_400_, kN/m^2^, kilonewton per square meter) calculated using the Mohr-Coulomb equation.

	Dirt		Synthetic	
Variable	Racetrack	Laboratory	Racetrack	Laboratory
c (N/m^2^)	4,162	1,493	2,040	2,244
φ (°)	33.3	40.2	38.2	39.6
R^2^	0.97	0.99	0.89	0.99
τ_400_ (kN/m^2^)	267	340	317	333

## Discussion

The validity of measuring dynamic impact properties of racetrack surfaces in the laboratory was examined by comparing measured properties *in situ* (racetrack) and in the laboratory. Laboratory surface reconstruction was guided by racetrack Clegg Hammer deceleration measurements. Relevance of observed differences in the dynamic behavior of race surfaces between settings was determined by comparison with observed differences between the dirt and the synthetic surfaces. Differences between racetrack and laboratory measurements were small relative to differences observed between dirt and synthetic surfaces (except for properties that had very small surface type differences). Thus, laboratory reconstruction of racetrack surfaces resulted in acceptable replication of measured dynamic impact properties of race surfaces for the desired application. However, due to the limited number of surface materials and maintenance procedures evaluated in the present protocol, findings in this study cannot be extended to all dirt and synthetic surfaces and their various maintenance conditions.

Small differences were observed between laboratory and racetrack measurements for dynamic properties that may be related to racehorse injury [2], [17]. Setting differences in Z-force maximum, average load rate, and average stiffness were less than 6% of surface racetrack averages. These differences were ≤13% of *in situ* surface type differences. Laboratory tests were developed to assess surface type differences in a controlled setting. The racetrack surface reconstruction protocol reproduced measurements sufficient for the intended application.

The effects of surface, impact velocity, and impact angle on dynamic surface properties were very similar in the laboratory to those observed at the racetrack [10]. The dirt surface, higher impact velocities, and vertical impacts generally resulted in harder dynamic surface properties. However, there was a some loss of ability to observe differences between levels within factors. For example, 1 of 5 deformation properties (elastic deformation) was significantly different between surfaces in laboratory tests, whereas 4 were significantly different between surfaces *in situ* (racetrack).

Multiple impact tests can be performed on the same constructed surface in the laboratory with appropriate surface preparation. In the present study, measurements from two sets of impact tests (interspersed with harrowing) on each reconstructed laboratory surface were not significantly different between sets. This result coincides with previous studies [9]–[11] that have shown the importance of maintenance for preventing surface hardening. Future reconstructions using the current protocol would allow for multiple impact tests of varying speed, angle, mass, etc. Alteration of these factors may allow for application of these techniques to other equine breeds and/or disciplines. These studies could require protocol modifications for differences in surface materials, and hoof mass and dynamics during different equine locomotor tasks.

Although Clegg Hammer impact deceleration measurements appeared useful for layered reconstruction of race surfaces in the laboratory, correlations between TTD and Clegg Hammer measurements at the racetracks were moderate to good for the synthetic surface and weak for the dirt surface. The better observed correlation for the synthetic surface may be related to lower variability across a range of impact velocities and two different impact masses (27.8 kg TTD, 4.5 kg Clegg Hammer). These findings are similar to TTD results from a previous study [10] where synthetic surface dynamic properties changed less with increased impact velocity compared to the dirt surface. The poor, negative correlation for dirt may be related to the lighter mass of the Clegg Hammer compared to the heavier TTD. Like other light impact devices, Clegg Hammer measurements are mainly used for assessing less stiff, upper layers of the surface [18], [19]. Conversely, the TTD penetrates these layers so measurements reflect harder sub-layers. The effect would be exacerbated for the dirt surface compared to the synthetic surface because dirt has a steeper gradation in hardness with increasing depth. The negative correlation for dirt also verifies the finding that different drop test masses can result in different conclusions for the same surface. Therefore, impact velocities and masses similar to the event of interest should be used [20]. In this context, impact devices greater than 4.5 kg (mass of the Clegg Hammer) should be used for Thoroughbred racetrack surface assessment.

Upper layer hardness is affected by lower layers. Clegg Hammer measurements from more superficial layers (0, 5 cm) of the racetrack surface were more highly correlated with TTD dynamic surface properties than those for deeper layers. For this reason, the deepest layer at the laboratory (15 cm) was often made harder than the racetrack average to ensure that upper layers achieved the desired hardness. Note that the Clegg Hammer is purported to analyze up to about 15 cm in depth [14], and these lower layers contribute to upper layer hardness. Track maintenance crews have recognized the effect of lower layers on surface properties, demonstrated by scheduled harrowing of the surface more than 10 cm deep.

Laboratory TTD measurements more closely approximated dirt racetrack measurements, compared to synthetic. Both surfaces exhibited more plastic behavior than elastic behavior [10]. Elastic behavior was more difficult to replicate in the laboratory, partly because laboratory surface reconstruction was guided by hardness measurements, rather than time or rate related measurements. Greater disparities in synthetic surface measurements may be due to greater elastic behavior not replicated in the laboratory.

Moisture content discrepancies between laboratory and racetrack settings likely contributed to some differences in dynamic surface properties between settings. Dirt surface dynamic properties are affected by moisture content [3]. The uppermost dirt layer (0 cm) extracted from the racetrack had a moisture content of 10–11%, and this moisture content was used in the laboratory. However, the average 0 cm depth moisture content at the dirt racetrack across all locations was 8.8% [10]. Under a previous relationship established between TTD peak deceleration and moisture content at a dirt track [3], this moisture content difference would make less than a 2% difference in peak force measurements. Similarly, lower layers of the synthetic surface in the laboratory had greater moisture contents than the racetrack average. The surface material extracted from the racetrack originally had 12% moisture content at 15 cm depth, but moisture migrated during construction to the drier 10 cm depth layer above (the 10 cm layer moisture content increased after each reconstruction). The lower layer moisture content differences likely had a minimal effect on measurements.

Setting differences in synthetic surface dynamic properties may be attributed to temperature differences. Synthetic surface properties are affected by temperature because their waxes undergo melting and softening at operating temperatures common to racetracks [12]. Although the effect of changes in temperature on horse race times and wax properties has been reported [21], the effect of temperature on overall dynamic surface properties is unknown. Upper layers of synthetic surface at the laboratory were about 3–6°C cooler than the racetrack average. Therefore, this temperature difference may have made the wax, as well as the overall surface, slightly harder than *in situ*.

Several reconstruction factors likely affected replication of racetrack dynamic surface properties in the laboratory. During reconstruction, it was more difficult to achieve compaction of the synthetic surface because of its composition. The additives and composition of the synthetic surface resisted the settling of particles during vibration, and thus impeded compaction efforts. Overall, laboratory reconstruction and harrowing were simplified as surfaces were constructed to be level, not graded like the racetracks, and the speed and force of harrowing did not fully replicate harrowing performed by tractors. Furthermore, laboratory surfaces were built in one day. Racetrack surface behavior is the result of different combinations of surface harrowing, water application, environmental factors, and horse traffic that occur over much longer periods of time. These differences could have affected surface cushion hardness and density. The surface cushion in the laboratory was mostly harder than that at the racetracks as shown by upper layer (0 cm) Clegg measurements (larger decelerations and smaller deformations). More specifically, cushion hardness could have affected time of detection of the 40 N force threshold for determination of impact initiation, and thus the velocity at impact initiation. Initiation would have been detected earlier in free fall for a harder cushion, which would explain lower impact velocities observed in the laboratory. Better replication of the surface cushion layer could decrease differences in deformation and time-related measurements.

Quasi-static shear vane measurements were not well replicated for the dirt surface. Simple racetrack measurements that capture the rate-dependent and horizontal surface properties in addition to the Clegg Hammer deceleration values may enhance the laboratory surface reconstruction guide used in the present study. Future studies should further explore horizontal properties of equine race surfaces.

Findings in the current study are limited to the specific racetrack surfaces, materials and environmental conditions examined. The behaviors of materials and reconstructed surfaces are expected to be non-linear and highly affected by a multitude of conditions (temperature, moisture, material components, compaction, etc). The current study did not address reproducibility of other surfaces or alternative reconstruction techniques. However, the study did provide the first objective data on racetrack surface mechanical behavior within a scaled, controlled laboratory setting, as well as evidence for the potential usefulness of this approach in the evaluation of racetrack surfaces. This approach is particularly valuable when considering the $8–10 million cost of replacing a single racetrack surface in the US. Changing racetrack surfaces in order to empirically evaluate conditions is not economical or practical. Therefore, this study is considered an initial step in the development of methodology for evaluating the properties of racetrack surfaces as a structure composed of surface material layers.

Important relationships between TTD and instrumented horse measurements have not yet been quantified. When horse hoof acceleration and TTD (36.4 kg impact mass, <1.6 m/s impact velocity) measurements from a dirt surface were fitted with polynomial equations, nonlinear relationships were found that were dependent on the horse gallop speed [3]. In the present study, the TTD had an effective mass and range of impact velocities more appropriate for simulating hoof strike at the fast trot or slow gallop [9], but relations between these TTD measurements and horses have not yet been specifically examined. Future studies should further characterize the link between hoof impact testing devices and racehorse hoof impacts.

In summary, equine racetrack surfaces were successfully reconstructed in the laboratory by replicating racetrack Clegg Hammer deceleration measurements throughout the depth of the surface reconstruction. While exact replication of all properties was not achieved, setting differences observed between laboratory and racetrack values for dynamic surface properties central to equine injury prevention were small, particularly relative to differences between dirt and synthetic surfaces *in situ*. The laboratory method used in the present study was useful for the objective evaluation of dynamic properties of racetrack surfaces in a controlled environment. Impact measurements derived from light, portable instruments may lead to different conclusions, compared to measurements from heavier devices designed to simulate a racehorse’s hoof impacting at the gallop.
