# Correction: Validation of a Laboratory Method for Evaluating Dynamic Properties of Reconstructed Equine Racetrack Surfaces

**DOI:** 10.1371/journal.pone.0177213

**Published:** 2017-05-03

**Authors:** Jacob J. Setterbo, Anh Chau, Patricia B. Fyhrie, Mont Hubbard, Shrini K. Upadhyaya, Jennifer E. Symons, Susan M. Stover

The majority of the in text citations are incorrect throughout the the paper. The first in text citation is correct.

The reference to [1] in the third sentence of the introduction should be a reference to [2].

All subsequent in text citations must be shifted up by one. Thus all references in the original article to [2] should be to [3], all references in the original article to [3] should be to [4], etc.
